# Canine pulmonary adenocarcinoma tyrosine kinase receptor expression and phosphorylation

**DOI:** 10.1186/1746-6148-10-19

**Published:** 2014-01-14

**Authors:** Evan T Mariotti, Christopher Premanandan, Gwendolen Lorch

**Affiliations:** 1 Department of Veterinary Clinical Sciences, College of Veterinary Medicine, The Ohio State University, Columbus, OH 43210, USA; 2 Department of Veterinary Biosciences, College of Veterinary Medicine, The Ohio State University, Columbus, OH 43210, USA

**Keywords:** Canine, Epidermal growth factor receptor, Lung cancer, Platelet-derived growth factor receptor, Tyrosine kinase receptors

## Abstract

**Background:**

This study evaluated tyrosine kinase receptor (TKR) expression and activation in canine pulmonary adenocarcinoma (cpAC) biospecimens. As histological similarities exist between human and cpAC, we hypothesized that cpACs will have increased TKR mRNA and protein expression as well as TKR phosphorylation. The molecular profile of cpAC has not been well characterized making the selection of therapeutic targets that would potentially have relevant biological activity impossible. Therefore, the objectives of this study were to define TKR expression and their phosphorylation state in cpAC as well as to evaluate the tumors for the presence of potential epidermal growth factor receptor (EGFR) tyrosine kinase activating mutations in exons 18–21. Immunohistochemistry (IHC) for TKR expression was performed using a tissue microarray (TMA) constructed from twelve canine tumors and companion normal lung samples. Staining intensities of the IHC were quantified by a veterinary pathologist as well as by two different digitalized algorithm image analyses software programs. An antibody array was used to evaluate TKR phosphorylation of the tumor relative to the TKR phosphorylation of normal tissues with the resulting spot intensities quantified using array analysis software. Each EGFR exon PCR product from all of the tumors and non-affected lung tissues were sequenced using sequencing chemistry and the sequencing reactions were run on automated sequencer. Sequence alignments were made to the National Center for Biotechnology Information canine EGFR reference sequence.

**Results:**

The pro-angiogenic growth factor receptor, PDGFRα, had increased cpAC tumor mRNA, protein expression and phosphorylation when compared to the normal lung tissue biospecimens. Similar to human pulmonary adenocarcinoma, significant increases in cpAC tumor mRNA expression and receptor phosphorylation of the anaplastic lymphoma kinase (ALK) tyrosine receptor were present when compared to the corresponding normal lung tissue. The EGFR mRNA, protein expression and phosphorylation were not increased compared to the normal lung and no activating mutations were identified in exons 18–21.

**Conclusions:**

Canine pulmonary adenocarcinoma TKRs are detected at both the mRNA and protein levels and are activated. Further investigation into the contribution of TKR activation in cpAC tumorigenesis is warranted.

## Background

The molecular characterization of canine lung cancer remains limited compared to the more comprehensive evaluation of human pulmonary adenocarcinoma (hpAC). A few similarities between the human and canine pulmonary adenocarcinoma (cpAC) molecular signatures have been identified and include the presence of Kirsten rat sarcoma viral oncogene mutations and overexpression of the epidermal growth factor receptor (EGFR) [1-3]. If canine lung tumors could be classified into distinct molecular subsets based on genomic alterations, use of targeted therapies may improve clinical outcomes.

Tyrosine kinase receptors (TKRs) are a family of cell-surface receptors that signal primarily through tyrosine phosphorylation events [4]. Many TKRs have been identified as key regulators of oncogenesis. Alterations in TKRs result in aberrant activation of their downstream intracellular signaling pathways that are linked to cancer growth, cell migration, cell-cycle control, cell survival, angiogenesis and metastasis. TKRs may become dysregulated by several oncogenic mechanisms including mutations, protein overexpression and increased gene copy number. The established dysregulation of TKRs and their contribution to lung cancers has continued to drive the development of drugs that block or attenuate TKR activity.

The EGFR is a TKR that has been identified as an oncogenic driver in certain subsets of human non-small cell lung cancer (hNSCLC). Approximately 60% of hpAC express EGFR. As such, EGFR has become an important therapeutic target for the treatment of these tumors. A range of somatic mutations in the tyrosine kinase domain of EGFR have been identified and are characterized by short deletions in exon 19, point mutations in exons 19 and 21, single nucleotide substitutions that may occur throughout exons 18 to 21, in-frame duplications and/or insertions predominately in exon 20 and rarely, an exon 22 mutation. Short deletions in exon 19 and an L858R point mutation in exon 21 account for greater than 85% of the mutations. Mutations in the EGFR tyrosine kinase domain result in destabilization of its conformation and constitutive kinase activity which provides continuous activation of its downstream signaling pathways. Lung cancers harboring constitutively active mutant EGFR are exquisitely sensitive to EGFR tyrosine kinase inhibitors (TKIs) with response rates reported from 27-100% [5].

Treatment for hpAC is surgical resection and adjuvant cisplatin-based chemotherapy in combination with vinorelbine. Primary radiation therapy is used in patients with inoperable stage II disease or with patients that have operable tumors with medical contraindications to surgery. Selective patients may benefit from molecularly-targeted therapy used in combination with chemotherapy, as a single first line-agent or maintenance chemotherapy. The results of standard treatment are poor as the 5-year relative survival rate varies depending on the stage at diagnosis from 49% to 16% to 2% for patients with local, regional and distant stage disease, respectively [6]. Chemotherapy for the dog has been largely unrewarding, with minimal responses to vindesine, cisplatin, and partial responses to vinorelbine [7,8]. No responses were seen in dogs with primary cpAC treated with the inhaled antineoplastic drugs paclitaxel or doxorubicin [9,10]. A dog with the diagnosis of primary bronchogenic carcinoma was treated with the TKI, toceranib, and was reported to have stable disease of 34+ weeks [11].

As the molecular profile of cpAC has not been well-characterized, identification of therapeutic targets that would potentially have relevant biological activity is impossible. Therefore, the objectives of this study were to define TKR expression and their phosphorylation state in cpAC as well as to evaluate the tumors for the presence of potential EGFR tyrosine kinase activating mutations in exons 18–21.

## Results

### Sample demographics

Twelve cases of cpAC (1 necropsy sample and 11 surgical samples) met inclusion criteria. The mean age at presentation was 11.2 years (median, 11.3; range, 8.1-15 years). Eight of the dogs were castrated males and four were spayed females. The most represented breeds were Basset Hound, Miniature Schnauzer, Labrador Retriever (two cases each), and a single case from a Border Collie, Boxer, Pomeranian, Scottish Terrier and mixed breed. Fifty percent of the dogs (6/12) were classified as large breeds, 25% (3/12) were medium breeds and 25% (3/12) were small breeds.

### Histopathology

Tumors classified as adenocarcinomas or the recognized subtypes were papillary 5/12 (47%), bronchioloalveolar carcinoma 6/12 (50%) and acinar 1/12 (8%). Hematoxylin and eosin (H&E) stained sections were used to determine histologic differentiation. Tumors varied in their degree of differentiation with 67% characterized as well-differentiated, 25% as moderately differentiated and 8% as poorly differentiated. Companion lung tissue classified as “normal” by clinical and histological appearance was present for all 12 cases.

### Selected receptor tyrosine kinase mRNA expression in cpAC biospecimens

**Figure 1:**
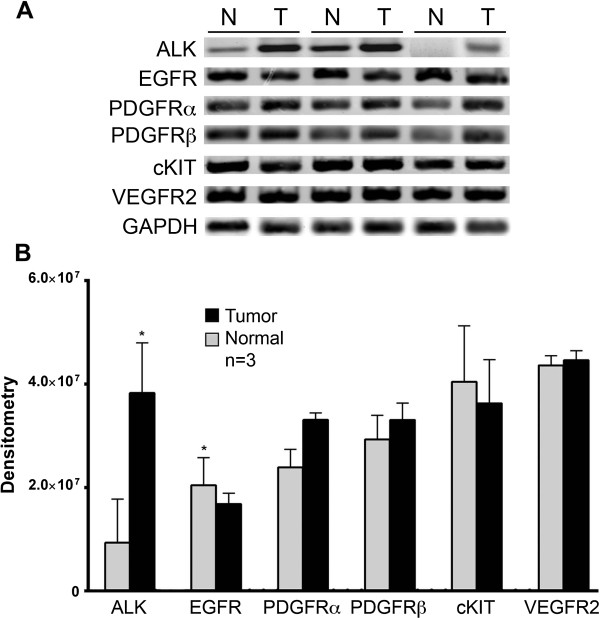
**Relative TKR mRNA expression levels in canine normal lung tissue and companion lung cpAC biospecimens from three dogs. (A)** Reverse transcriptase TKR cDNA transcripts in normal lung (N) and lung cpAC (T). GAPDH serves as a loading control. **(B)** Densitometry: the levels of tumor TKR cDNA normalized to total normal lung and GAPDH. Error bars, mean ± SEM. *EGFR and ALK cpAC/normal of three dogs: *P* < .05.

Reverse transcriptase-polymerase chain reaction (RT-PCR) was used to identify the presence of cpAC mRNA of fifteen different receptor and cytoplasmic tyrosine and serine/threonine kinases (Table 1). Message for all the selected TKRs and downstream serine and threonine kinases in Table 1 was present in all of the twelve dog’s tumors and companion normal lung biospecimens. Densitometry of relative RT-PCR EGFR tumor transcripts were significantly decreased compared to normal lung EGFR message, whereas transcripts for ALK were significantly increased in tumors compared to the corresponding normal tissue ALK message (n = 3) (Figure 1).

**Table 1:** Canine RT-PCR primer sets for selected tyrosine kinase receptors and signaling node kinases

**Canine-specific primers used for EGFR PCR amplification of the exon gDNA**		
	** Forward**	** Reverse**
*Exon 18*	5′-TGAGCATTGCGGCAGTTGCT-3′	5′-ACACCCGGAAGTCATGGAAC-3′
*Exon 19*	5′-GAGTGCACAGCTCTGCTCAAG-3′	5′-GCTATCCGGGCCTGTGGACAAG-3′
*Exon 20*	5′-CTCTGAAGCTTTCCTCTCCCC-3′	5′-GCGAAGTACCTGCCCTACTTT-3′
*Exon 21*	5′-GGCATGAACTACCTGGAAGACCG-3′	5′-CTGTGATCTTGACGTGCTGCG-3′
**Primers used for RT-PCR**		
**Primer set**	** Forward**	** Reverse**
*AKT*	5′-TGCTTAAGAAGGACCCCAAGC-3′	5′-GCTGGTCCAGTTCGAGGGA-3′
*ALK*	5′-CTGTATCGGGGTGAGTCTGC-3′	5′-CAGGGCCTGGACAGGTTAAG-3′
*c-KIT*	5′-GATGGCCCCTGAGAGCATTT-3′	5′-GCCTTTTCAGGGGATCAGCA-3′
*EGFR*	5′-TGGTCCTGGGGAATTTGGAA-3′	5′-GGTTATTGCTGAAGCGCACA-3′
*ErbB2/HER2*	5′-CCCGAGACCCACCTGGATA-3′	5′-CAGGGCGTAGTTGTCCTCAA-3′
*ErbB3/HER3*	5′-CGAGGAGGTGTCTGTGTGAC-3′	5′-CTTGTAGATGGGGCCCTTGG-3′
*FGFR1*	5′-TTTTCAGCCTTGAAAGCACCATAA-3′	5′-CAGCTGAATGTGCTGGTCGC-3′
*FGFR4*	5′-CTACCTCCTGGGCATCAAGC-3′	5′-GAACTTGCACTCCTCGGTGA-3′
*FLK-1/VEGFR2*	5′-CTAGGTAAGCCTCTTGGCCG-3′	5′-ACAATCACCATGAGTGGCCC-3′
*GAPDH*	5′-GCCTGGAGAAAGCTGCCA-3′	5′-AGGTGGAAGAGTGGGTGTCA-3′
*MAPK*	5′-AGGCTGTTCCCAAATGCTGA-3′	5′-CCTTGGGCAGGTCATCTAGC-3′
*PDGFRα*	5′-TGATTGAGAGGCTGGCACAC-3′	5′-AATGACCGTCCTGGTCTTGC-3′
*PDGFRβ*	5′-GGCCCGTTCAGGTGAGAAAA-3′	5′-GAGTTTGGGGTGTCTTGGCT-3′
*RET*	5′-AGCAGGATACACGACCGTTG-3′	5′-GTCCCGATGGACAAGCTTCA-3′
*ROS-1*	5′-CTGTGAGAGCCAGTTCACCC-3′	5′-ACATCACCTTTCACGTCGCT-3′
*RPS6*	5′-GCTAAGCGGAAGTCGGGTC-3′	5′-TGGGGAAGCCTTGTTTGTCA-3′

### IHC quantification of tyrosine kinase protein expression in TMA cpAC and normal lung biospecimens

**Figure 2:**
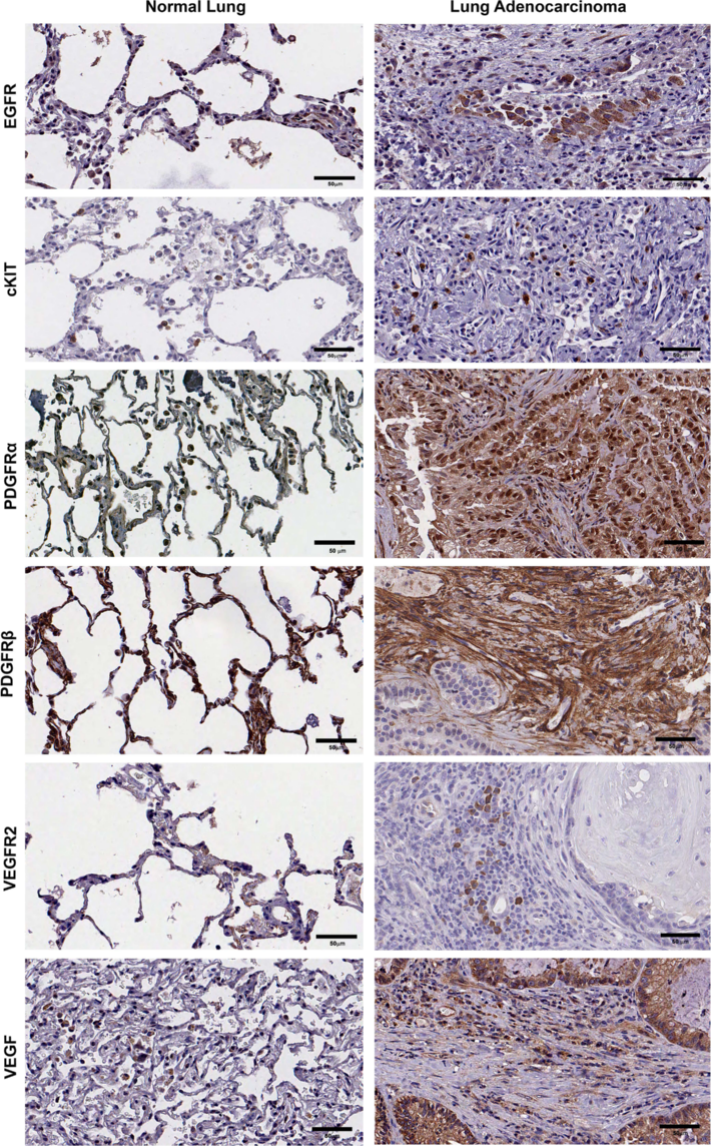
**Representative micrographs of immunohistochemistry for selected TKRs and VEGF expression in TMA cores of normal lung and cpAC samples.** The tissues were probed with the antibodies for EGFR, cKIT, PDGFRα, PDGFRβ, VEGFR2 and VEGF proteins. Left set of panels: Representative images of positive immunoreactivity for the TKRs and growth factor as shown by the brown staining in normal lung. Right set of panels: Representative images of positive immunoreactivity for the TKRs and growth factor as shown by the brown staining in neoplastic cells and/or stroma of the adenocarcinoma tissue. x 40; bar 50 μm.

Canine pulmonary adenocarcinoma tissue microarrays were used to discover putative TKR tumor expression phenotypes. All cpAC and normal lung core specimens were available for IHC analysis. Subjective classification of immunopositivity for EGFR localization in normal lung was determined to be in cytoplasm of the bronchial and vascular smooth muscle, basal layer of the bronchial epithelium, alveolar epithelium and the cytoplasm of alveolar macrophages. cKIT positive staining of the normal lung was considered moderate and found in cytoplasm of alveolar macrophages. Normal lung PDGFRα immunopositivity was located in the nucleus and cytoplasm of alveolar epithelium and macrophages, whereas the bronchiolar epithelium demonstrated moderate diffuse staining of the cell membrane, cytoplasm and nucleus. Strong immunopositivity for PDGFRβ was predominately in the pulmonary interstitium, pneumocytes and peribronchiolar fibroblasts of the normal pulmonary biospecimens. Faint VEGFR2 positive reactivity was present in interalveolar fluid of the normal lung as well as found on either side of the pneumocytes, while positive staining for the ligand, VEGF, was present in the cytoplasm of bronchiolar epithelium and alveolar macrophages (Figure 2, left panels).

Canine pulmonary adenocarcinoma TMA cores were subjectively graded by a board-certified pathologist using a semiquantitative scoring scale for intensity and localization of immunolabeling. The slides were evaluated blinded. EGFR staining was present in the tumor cell cytoplasm from every dog. Positivity for VEGFR2 was present in 50% (6/12) of the patients’ tumors, whereas 100% of the tumors were positive for VEGF. Only 25% (3/12) of the patients’ tumors were positive for c-KIT. All tumors were positive for PDGFRα immunoreactivity and had the greatest overall percentage of positive staining neoplastic cells. Two cpACs had faint stromal staining for VEGF and PDGFRα whereas; PDGFRβ had distinct strong stromal tumor staining in 92% (11/12) with faint positive cytoplasmic staining and rare membranous staining of the neoplastic cells in 75% (9/12) (Figure 2 and Table 2).

**Table 2:** Subjective scoring of TKR immunoreactivity in cpAC biospecimens from 12 dogs

**TKR**	**No staining n(%)**	**0 n(%)**	**1 n(%)**	**2 n(%)**	**3 n(%)**	**Stromal staining n(%)**	**Predominant localization**	**Stained areas**
EGFR	**0**	**2 (17)**	**3 (25)**	**4 (33)**	**3 (25)**	**0**	**C**	**C, M**
VEGFR2	**6 (50)**	**5 (42)**	**0**	**1 (8)**	**0**	**0**	**C**	**C, M**
VEGF	**0**	**1 (8)**	**6 (50)**	**2 (17)**	**3 (25)**	**2 (17)**	**C**	**C, S, N**
c-KIT	**9 (75)**	**1 (8)**	**2 (17)**	**0**	**0**	**0**	**C**	**C, M**
PDGFRα	**0**	**0**	**1 (8)**	**2 (17)**	**9 (75)**	**2 (17)**	**C, N**	**C, S, N**
PDGFRβ	**1 (8)**	**1 (8)**	**4 (33)**	**3 (25)**	**1 (8)**	**11 (92)**	**C, S**	**C, S, M**

Result numbers (n) and percentages (%) represent number of cpAC- affected dogs. Percentage of neoplastic cells staining positive were scored as follows: 1-5% = **0**, 5-25% = **1** 26-50% = **2** and >50% = **3**. C: cytoplasmic, M: membranous, N: nuclear, S: stromal. No staining represents number and percentage of cpAC-affected dogs that have no evidence of any positive immunoreactivity.

**Figure 3:**
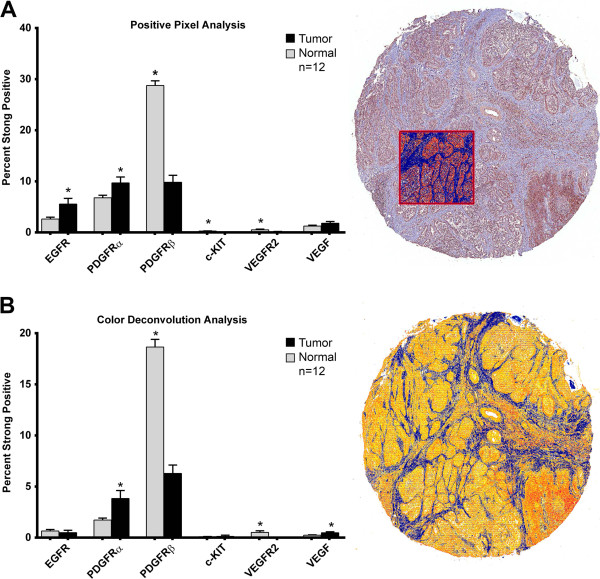
**Quantification of TKR immunopositivity in normal and tumor TMA cores using algorithm analysis software. (A)** Positive pixel analysis is a digitalized pixel count in a user defined region (red square) of each core as shown in the visual representation of the TMA core for PDGFRα analysis (right). The bar graph represents the percent strong positive pixels for each TKR and VEGF (left). The fraction of strong stained pixels is the number of total pixels (blue and red) minus the negative pixels (blue) in the markup image of the TMA, divided by the number of total stained pixels. **(B)** Color deconvolution analyses of IHC digitalized images accounted for different staining densities of the entire core. The intensity range markup core of PDGFRα analysis (right) shows intensity ranges as weak positive staining (yellow), medium (orange), strong positive (red) and negative staining (blue). The biospecimen percent of positive pixels strongly stained for TKRs and VEGF are represented in the bar graph on the left. Each bar represents the mean positivity of 36 tumor (black) and 36 normal (grey) TMA cores from 12 dogs. Error bars, mean ± SEM. *cpAC *vs.* normal: *P* < .05.

All 12 normal and tumor tissue sets of TMA cores were used for IHC quantification. The greatest IHC percent positivity was seen for the TKR, PDGFR family. A statistically significant increase in the percentage of strong staining for PDGFRα was present in cpAC compared to normal lung as determined by both the positive pixel or color deconvolution algorithms. Both algorithms also quantified a statistically significant increase in the percentage of strong staining PDGFRβ and VEGFR2 in the normal lung cores compared to the cpAC cores (Figure 3A-B). A greater percentage of strong staining intensity for the growth factor VEGF was present in cpAC biospecimens, however; the cpAC VEGF quantification only reached statistical significance when compared to the normal lung cores using the color deconvolution algorithm. Quantification of the percentage of EGFR strong immunopositivity was significantly increased in the cpAC using the positive pixel algorithm; however, when the measurement of percent strong positive staining considered the entire tissue core using color deconvolution algorithm, the normal lung cores had a slightly greater positivity compared to the cpAC. Both algorithms quantified c-KIT staining as negligible in all tissues.

### Relative level of receptor and cytoplasmic tyrosine and serine/threonine kinase phosphorylation in cpAC biospecimens

**Figure 4:**
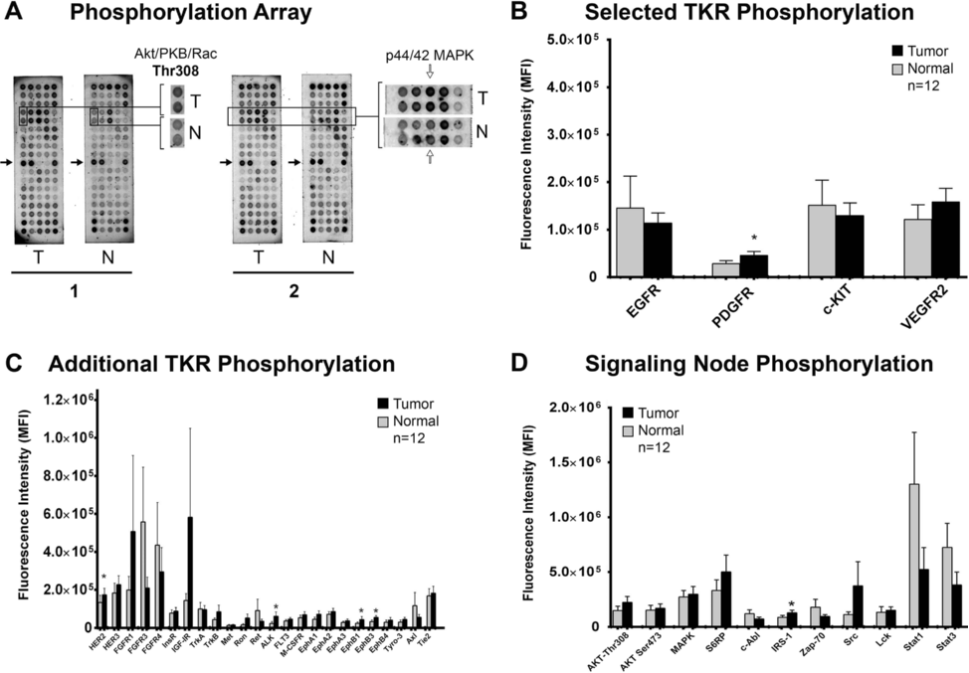
**Quantification of basal TKR and signaling node phosphorylation of cpAC and normal lung tissue. (A)** Representative captured fluorescent image of a slide with four phosphorylation array profiles from paired normal and tumor protein lysates of two dogs. Differences between tumor and normal tissue fluorescence intensities for Akt/PKB/Rac at Thr308 are highlighted in the array set from the first dog, whereas differences between tumor and normal tissue fluorescence intensities for p44/42 MAPK are highlighted in the array set from the second dog (white arrows). The black arrow represents one row of the array spotted with three positive (black spots) and two negative (non-colored spots) controls. The entire array has 10 positive controls. **(B-D)** The protein array measured the phosphorylation status of the selected TKRs that were evaluated using IHC as well as 24 additional TKRs and 11 downstream signaling nodes. The phosphorylation measurement of PDGFR includes pan phosphorylation of both the alpha and beta forms of this receptor. Spot intensities were quantified using ImageQuant™ TL array analysis software. Each bar represents the mean fluorescence intensities obtained and therefore phosphorylation of 12 tumor (black) and 12 normal (grey) normal lung lysates from each dog. Error bars, mean ± SEM. *cpAC *vs.* normal: *P* < .05.

To begin to identify which kinase families and downstream signaling nodes are expressed and activated in cpAC, we evaluated the phosphorylation state of 28 TKRs and 11 downstream signaling cascades using a slide-based antibody array. The phosphorylation status of the TKRs and signaling nodes were determined in the normal lung tissue and tumor for each dog (Figure 4A). The overall mean phosphoTKR and signaling node fluorescence intensities were greater in the cpAC when compared to normal lung, with the cpACs having increased phosphorylation of 75% (21/28) of the TKRs and 64% (7/11) of the signaling nodes (Figure 4B-D). When considering TKR families, the strongest cpAC fluorescence intensities were identified in the EGFR and Insulin R families. Phosphorylation of the fibroblast growth factor receptor, (FGFR) family was the greatest in the normal lung with phosphoFGFR3 and FGFR4 accounting for the increased intensities. Statistically significant increases in the phosphorylation of cpAC TKRs were found for PDGFR, HER2, ALK, EphB1 and EphB3 and the signaling node insulin substrate receptor-1. A summary of the percentage of biospecimens that had TKR phosphorylation are presented in Table 3. All tumor and normal lung biospecimens had positive fluorescence and therefore phosphorylation of all serine and threonine signaling nodes.

**Table 3:** Percentage of cpAC and normal lung biospecimens with TKR phosphorylation

**TKR**	**cpAC n(%)**	**Normal lung n(%)**
EGFR	**11 (92)**	**10 (83)**
HER2	**12 (100)**	**11 (92)**
HER3	**12 (100)**	**12 (100)**
FGFR1	**10 (83)**	**9 (75)**
FGFR3	**10 (83)**	**10 (83)**
FGFR4	**10 (83)**	**9 (75)**
InsR	**11 (92)**	**12 (100)**
IGF-IR	**12 (100)**	**12 (100)**
TrkA/NTRK1	**12 (100)**	**11 (92)**
TrkB/NTRK2	**12 (100)**	**11 (92)**
Met/HGFR	**10 (83)**	**10 (83)**
Ron/MST1R	**11 (92)**	**10 (83)**
Ret	**10 (83)**	**12 (100)**
ALK	**11 (92)**	**11 (92)**
PDGFR	**11 (92)**	**10 (83)**
c-KIT/SCFR	**6 (50)**	**11 (92)**
FLT3/Flk2	**7 (58)**	**11 (92)**
M-CSFR	**9 (75)**	**11 (83)**
EphA1	**11 (92)**	**12 (100)**
EphA2	**11 (92)**	**11 (92)**
EphA3	**11 (92)**	**11 (92)**
EphB1	**11 (92)**	**10 (83)**
EphB3	**12 (100)**	**10 (83)**
EphB4	**11 (92)**	**11 (92)**
Tyro-3/Dtk	**11 (92)**	**10 (83)**
Axl	**7 (58)**	**12 (100)**
Tie/TEK	**12 (100)**	**12 (100)**
VEGFR2/KDR	**6 (50)**	**11 (92)**

Phosphorylation of biospecimen protein lysate was determined by the presence of fluorescence that was quantified after normalization to the array spotted positive and negative controls for each sample. Each receptor was assayed in duplicates and the mean fluorescence for that receptor was recorded. n, represents the number of positive samples and (%) represents the percentage out of 12 total samples.

### Mutational profiling of EGFR

To begin to understand if EGFR expression and activation in cpAC could be related to altered EGFR tyrosine kinase signaling, genomic DNA for the EGFR exons, 18, 19, 20 and 21 from each cpAC and matched normal lung biospecimens was sequenced. No mutations were identified for any of the exons in either the non-affected or affected lung tissues of any of the patients.

## Discussion

In hNSCLC, TKRs frequently become mutated, overexpressed or become fusion genes as a result of chromosomal translocations. These alterations perturb cell behavior resulting in constitutive and aberrant activation of mitogenic cellular pathways. Oncogenic TKR drivers in hpACs include *EGFR* mutations*, ErbB2* insertions*, ALK, ROS1 and RET* fusions, and *MET* amplifications. The importance of the identification of patients with these aberrant TKRs has led to personalized small molecule inhibitor therapy and thus has improved progression-free survival (PFS) rates. The findings in this study parallels those found in hNSCLC as we demonstrate statistically significant increases in the phosphorylation of five TKRs and one downstream signaling node as well as increased TKR immunohistochemical expression for four TKRs in cpAC. Notably, increased phosphorylation of ErbB2 and ALK receptors were found in our cohort of cpAC biospecimens, which recapitulates the findings in hpACs. Gene amplification, epigenetic mechanisms and oncogenic viruses as causes of the increased TKR protein were not evaluated in the current study.

The EGFR is a TKR whose activation is crucial for the growth and survival of hpAC. Two studies to date have evaluated EGFR expression using IHC in cpACs tissues [1,12]. The localization of EGFR protein to the bronchial epithelium and submucosal glands of the normal lung parenchyma in the present study corresponds to the findings of previous studies [1,12]. However in our population of dogs, EGFR IHC positivity was also present in both the alveolar macrophages and alveolar epithelial cytoplasm. Although human alveolar macrophages produce EGF in a tissue and disease-specific manner [13,14], they do not have the EGFR receptor, suggesting that the antibody used to detect EGFR in this study may lack specificity to distinguish between the ligand and receptor as they do have protein sequence homology at the C-terminus [15,16]. Unlike human alveolar macrophages which only produce EGF, type II pneumocytes of adult rats produce EGF and express EGFR which use an autocrine mechanism that likely regulates pneumocyte differentiation and growth [17,18].

Semiquantitative evaluation of IHC indicated that all cpAC had immunopositivity for EGFR and therefore at least 1-25% of the neoplastic cells had EGFR staining. The percentage of dogs with neoplastic cell EGFR positivity in this study is similar to what has been previously reported. In a study that had 25 cases of cpAC, 80% of the tumors expressed EGFR and of the cpACs that had EGFR, the percentage of tumor cells counted as positive ranged from 20-100% [1]. Genetic alterations similar to those found in human EGFR exons were investigated by sequencing the tyrosine kinase domains of both the cpACs and the non-affected tissues for detection of deletions, mutations or single nucleotide polymorphisms in exons 18–21. Sequence analysis of the cpAC EGFR TKR domains did not identify any significant nucleotide substitutions. Additional genomic analysis of cpACs for the EGFR transforming C-terminal domain deletion mutants of exons 25 to 27 and exons 25 to 28 should be performed as these are designated as chemo-responsive to anti-EGFR therapy [19].

The statistically significant increased phosphorylation fluorescence intensity of the *EGFR* family member HER2 (*ErbB2*) raises the possibility of this receptor playing a pivotal role in malignant progression. ErbB2 uniqueness from its other family members is its inability to directly bind any known EGF family ligand and its permanent fixation in the constitutively active conformation. As such, ErbB2 heterodimers demonstrate increased potency in conveying extracellular signals [20]. ErbB3 does not have intrinsic catalytic kinase activity. The most powerful signaling heterodimer in the *EGFR* family is composed of ErbB2 and ErbB3 which functions as an oncogenic unit [21-23] capable of activating the PI3K/Akt pathway. Novel oncogenic *ErbB2* extracellular domain mutants have been identified in hpAC, which are activated by elevated C-terminal tail phosphorylation or by the formation of disulfide-linked dimers [24]. Cell lines that overexpress *ErbB2* extracellular domain mutants have been shown to become potently oncogenic and have increased cell motility [24]. The increased phosphorylation and therefore constitutive activation ErbB2 in cpAC provides rationale for detailed mechanistic evaluation.

Approximately 3 to 7% of hNSCLC tumors are characterized by rearrangement of the gene encoding anaplastic lymphoma kinase (*ALK*) most commonly with echinoderm microtubule-associated protein-like 4, resulting in constitutively active kinases with transforming capacity [25]. Tumors identified as having ALK fusion proteins have a dramatic therapeutic response to specific ALK inhibitors [26]. The small molecule inhibitor crizotinib has demonstrated high anti-tumoral activity, significantly higher response rate and longer PFS in *ALK*-positive defined lung cancers. ALK phosphorylation was significantly increased in our cpAC cohort, as were the cpAC ALK mRNA transcripts. As IHC represents a common method for detection of protein expression, we evaluated the TMA for immunostaining of ALK to validate the mRNA and phosphorylation results. Unfortunately, immunostaining for ALK was negative using the selected antibody and method on formalin fixed paraffin embedded samples of canine normal lung, cpAC and the three positive control canine tumors. However, IHC ALK staining was positive when used on hpAC tissue section known to have an ALK-fusion protein. IHC detection of ALK in hNSCLC tumors presents a significant challenge due to differential expression of ALK protein occurring at a low level and due to the performance of current available fluorescence in situ hybridization and IHC methods which lack sensitivity and reproducibility [27,28]. To improve IHC assay sensitivity, a novel, non-endogenous hapten, 3-hydroxy-2-quinoxaline and tyramide amplification into a DAB-HRP based assay has been developed and detects low levels as well as heterogeneous ALK protein expression in NSCLC TMAs but it is not commercially available [27]. The findings of increased cpAC ALK mRNA expression and phosphorylation as well as the trend toward increased cpAC phosphorylation of the downstream AKT and MAPK pathways, would provide reason for speculation that ALK may be a promoter of cpAC tumorigenesis.

The identification of significant increases in PDGFRα protein expression and phosphorylation in cpAC may represent a mechanism to promote angiogenesis [29,30]. PDGFRα –mediated paracrine signaling between tumor cells and stromal fibroblasts was found to be a principal mechanism for stroma recruitment and tumor growth especially when tumor cells are deficient in VEGF production [30]. The protein for the angiogenic growth factor, VEGF, was also increased in both quantitative TMA analyses as was the phosphorylation of VEGFR2, although not reaching statistical significance. The finding of a significantly increased VEGFR2 protein by IHC in the normal lung likely reflects the presence of vast vascular capillary plexus that surrounds alveoli. Only 20% of humans with NSCLC have increased tumor VEGFR2 expression which correlates with a highly angiogenic phenotype. Human NSCLC angiogenesis is triggered by a tumor VEGF/VEGFR2 autocrine feed-forward loop which amplifies VEFG secretion by tumor cells and when this loop is inhibited, the tumor changes to a proliferative phenotype that sensitizes tumor cells to MAPK inhibition [31]. Taken together, the data may suggest that cpACs have activated receptors that likely contribute to angiogenesis pathways for promotion of tumor growth and metastasis.

## Conclusions

An acknowledged limitation to this work is that it is descriptive in nature. The observed phosphorylation of the TKRs and downstream effectors needs to be further validated in cpAC cell lines that are known to express these receptors. This may be difficult as canine cpAC cell lines are scarce. Furthermore, protein expression by IHC does not directly correlate with a causative role in tumor growth and survival. The observed discrepancy between tumor IHC EGFR protein expression level could be due to the qualitative nature of IHC with the use of an EGFR antibody that lacked sensitivity for canine tissue, differences in sensitivities of the two digital positive pixel quantification algorithms, as well as, the subjective determination of the staining by the pathologist. Furthermore, the samples used for the tumor lysates were not microdissected and thus contain tumor, stroma and non-neoplastic cell infiltrates. When comparing receptor mRNA and protein levels in a tumor to “histologically normal lung” tissue within the same individual, it is possible that molecular pathology has preceded the development of morphologically identifiable changes through the “field of cancerization” effect. As such, the normal tissue may have been in a precancerous state diminishing the possibility of finding differences. Although we found no evidence of mutations or significant single nucleotide polymorphisms in the selected EGFR exons that were sequenced from the normal lung tissues when compared to the NCBI canine reference gene, we are unable to definitively state that the “normal” lung tissue was not in a molecularly significant pre-neoplastic state for every receptor evaluated in this study. The inability to detect statistically significant differences in many of the TKRs at the protein and phosphoprotein levels may have been affected by the power of the study. If differences in the number of the receptors between the normal lung and the cpAC were relatively large, the power would be less affected however, given the heterogeneity of tumors it may be that the number of primary cpACs used was not sufficient to find a statistically significant result. Finally, the multiplexed phosphorylation TKR assay is labeled for human phosphorylation receptor detection as only approximately 50% of the phosphotyrosine receptor antibodies have been validated for canine use. Thus, the use of this comprehensive screening array could potentially have a lower sensitivity and specificity in our patient population.

Nevertheless, this work establishes that cpAC TKRs are detected at both the mRNA and protein levels and are activated. Although this study analyzed a limited number of samples, the results expand our cpAC knowledge and indicate that much work is still needed in this field.

## Methods

### Case inclusion and exclusion criteria

The medical records of all dogs with a diagnosis of primary cpAC were reviewed to acquire signalment, clinical and pathological diagnosis data. All dogs with a clinical diagnosis of cpAC had H&E stained slides made from recuts of their formalin-fixed, paraffin embedded tissue blocks for evaluation by a third veterinary anatomic pathologist (CP). This pathologist’s evaluation was used to confirm the previous diagnosis of cpAC. Patients included in this study were diagnosed with cpAC and had been evaluated during a five-year period (2007–2012). Study inclusion criteria were a clinical and pathological diagnosis of primary lung cpAC, the patient was stable enough to undergo a lung lobectomy and consent had been given by the owner to collect tissue specimens for the biospecimen repository. Exclusion criteria included chemotherapy prior to tumor resection, evidence of other neoplasia, unstable clinical disease and lack of consent to collect tissues.

### Biospecimens

Canine pulmonary adenocarcinoma tissue specimens were obtained from cases that presented to The Ohio State University Veterinary Medical Center (OSU-VMC). Owner consent to allow tissue collection was obtained in accordance with the approved IACUC protocol. Samples were obtained during surgery and snap frozen in liquid nitrogen within 15 minutes of harvest and stored at −80°C. Tissue was also placed in formalin and processed for routine paraffin embedding for evaluation of H&E stained sections. Tissue was cataloged and banked in the OSU-VMC Biospecimen Repository. A total of 12 primary lung adenocarcinomas with paired normal lung tissues from the same dog were used. A de-identified human lung adenocarcinoma was obtained from the National Cancer Institute Cooperative Human Tissue Network Biospecimen Repository under The Ohio State Cancer Institutional Review Board approved protocol 2011C0112 and was used as a positive control for ALK gene translocation. Routine biomarker testing was performed for the presence of ALK translocation using fluorescent in situ hybridization with the FDA-approved Vysis ALK Break- Apart FISH Probe Kit (06 N38-020, Abbott Molecular, Del Plaines, IL) which is optimized for identifying and quantifying rearrangements of ALK gene from formalin-fixed, paraffin-embedded hNSCLC tissue specimens. The sample was considered positive as >25 cells out of 50 had positive fluorescence as validated by both a pathologist as well as semi-automated scanning imaging analysis software.

### RNA isolation and reverse-transcriptase polymerase chain reaction

Total RNA was isolated from dog lung tissue and cpAC tumors using the Absolutely RNA Miniprep Kit (Stratagene, La Jolla, CA) according to the manufacturer’s instructions. The quality and quantity of the RNA was determined spectroscopically using a NanoVue Spectrophotometer (GE Healthcare, Piscataway, NJ). TaqMan® Reverse Transcription kits (Applied Biosystems, Carlsbad, CA) were used to make cDNA for RT-PCR analysis of all transcripts of the genes. Primer sequences used for RT-PCR reactions are listed in Table 1. Gel electrophoresis was used to separate the amplified cDNA products according to size. Amplicons were resolved on a 1% agarose gel to visualize the products. Densitometry measurements of each cDNA band were determined. Data were normalized by determining the ratio of the target cDNA concentration to GAPDH to correct for differences in RNA quantity between samples. Each of the three dogs normal and tumor transcripts were evaluated in triplicate for each gene. ImageQuant™ TL Analysis software version 7 (GE Healthcare, Piscataway, NJ) was used for densitometric quantification.

### DNA extraction and PCR of EGFR

DNA from all dog lung tissue and cpAC tumors was prepared using the DNeasy Blood and Tissue Kit (Qiagen, Valencia, CA) according to the manufacturer’s instructions. General PCR conditions used to amplify *EGFR* were an initial denaturation at 95°C for 2 min, 32 cycles of denaturation at 97°C for 30 s, annealing at 65°C for 60 s, and extension at 72°C for 90 s followed by elongation at 72°C for 7 min and terminated at 4°C. All amplifications used a high fidelity polymerase (Platinum Taq HiFi Polymerase, Invitrogen, Carlsbad, CA). Primers are listed in Table 1.

### Sequencing and sequence alignment

Standard PCR was used to generate high fidelity *Taq* polymerase-amplified PCR products. Amplicons were resolved on a 1% agarose gel to visualize the products. The resolved PCR products were extracted from the gel, purified using QIAquick PCR Purification kit (Qiagen, Germantown, MD) and sent for sequencing. Each exon and PCR product was sequenced using BigDye™ Terminator Cycle Sequencing chemistry (Applied Biosystems, Carlsbad, CA). Sequencing reactions were run on an Applied Biosystems 3730 DNA Analyzer automated sequencer. Sequence alignments were made to the reference sequence NCBI: EGFR epidermal growth factor receptor [*Canis lupus familiaris* (dog)]Gene ID: 404306, updated on 19-Oct-2013) using the ClustalW procedure in DNASTAR Software v MegAlign™ 5.06, Madison, WI.

### Tissue microarray and immunohistochemistry

An anatomic pathologist (CP) reviewed the H&E stained slides for each dog and selected three representative sites of the cpAC tumor and normal lung to be used for the construction of the tissue microarray (TMA). The corresponding sites from the H&E slides were then matched to the paraffin embedded tissue block, 2 mm cores were extracted and placed into the predetermined sites on the TMA recipient blocks. A computer-controlled autostainer as above (Dako, model S3400, Carpinteria, CA) was used to carry out the immunostaining. Immunohistochemistry staining was performed for EGFR (Santa Cruz Biotechnology, Inc., #sc-03; 1:500) [32,33], canine stem cell factor receptor (CD117/c-kit; Dako #A4502; 1:250) [34], platelet-derived growth factor receptor alpha (PDGFRα; Santa Cruz Biotechnology, Inc., #sc-338; 1:50) [35], platelet-derived growth factor receptor beta (PDGFRβ; Biogenex, #Nu483-uc; 1:200) [36], vascular endothelial growth factor receptor two (KDR/Flk-1/VEGFR2; Santa Cruz Biotechnology, Inc., #sc-6251; 1:50) [37,38], vascular endothelial growth factor (VEGF; Santa Cruz Biotechnology, Inc., #sc-152; 1:200) [39] and anaplastic lymphoma kinase (ALK; Cell Signaling Technology, #3633; 1:50). Slides were rinsed in wash buffer and incubated with the secondary biotinylated goat anti-rabbit or anti-mouse antibodies diluted to 1:200 in protein block for 30 min and rinsed in wash buffer. The slides were incubated for 30 min in an avidin/biotin-based peroxidase system to allow detection of biotinylated antibodies (VECTASTAIN® Elite_®_ ABC System, Vector Laboratories INC, Burlingame, CA). Tyrosine kinase receptor and VEGF positive controls for the TMA IHC were single tissue cores of canine melanoma, prostate adenocarcinoma and apocrine gland anal sac adenocarcinoma. The selected TKRs and VEGF have been previously identified in these canine tumor types or from tumor-derived cell lines [40-43]. Canine lung served as the positive control for expression of EGFR protein [1]. Negative controls were irrelevant isotype matched antibody at matched dilutions.

### Immunohistochemistry quantification and scoring

Quantification of IHC staining was performed on digitalized TMA slides by Aperio ScanScope XT, (Aperio Technologies, Vista, CA) and by semiquantitative grading by a veterinary pathologist. Image analysis was performed using two different Aperio algorithms. The first IHC quantification analysis was done using positive pixel count v.9 algorithm counts and quantifies the amount of stain of a specific color set by the user base on the HIS (hue, saturation, intensity) color model. The algorithm output provided a number of 1+, 2+ and 3+ intensity positive pixels and the number of total pixels in each annotated layer. Total number of pixels consisted of positive and negative pixels excluding white area of the virtual slides (i.e., the center of bronchi and alveoli). The results are reported as positivity which is the total number of positive pixels/total number of pixels. Briefly, positive pixel counts (PPC) were obtained for a square area of 393598 μm^2^ at a magnification of 20X for all the tumor and normal lung cores. For each dog, the PPC obtained from each of the three tumor cores was normalized to the PPC from each of the three normal lung cores. The normalized PPC from each of the 12 dogs was used to calculate the overall IHC PPC value for the receptor or growth factor. Color deconvolution was the second algorithm used for IHC quantification. This algorithm separated the image into channels based on normalized optical density and then overlapped these channels and evaluated the entire tissue core area of each sample. Normalized optical density was derived from the control DAB stained and unstained lung tissues used in each individual automated IHC processor staining run.

The subjective quantitative scoring of positive or negative immunoreactivity of cells and stroma within the cores was performed by a veterinary pathologist. Percentage of neoplastic cells staining positive were scored as follows: 1-5% = 0, 5-25% = 1, 26-50% = 2 and >50% = 3. Location of staining was also noted as cytoplasmic (C), membranous (M), nuclear (N), and stromal (S). Specimens that had no IHC staining were designated as having “no staining”.

### Protein isolation

Forty grams of either whole lung tissue or tumor were placed in a gentleMACS™ M tube (Miltenyi Biotec, Auburn, CA), with 400 μL of 1x cell lysis buffer (Cell Signaling, Danvers, MA), 1 mM PMSF and protease inhibitors (Halt protease inhibitor cocktail kit, Pierce, Rockford, IL, USA), homogenized with a gentleMACS™ dissociator (Miltenyi Biotec, Auburn, CA) and then centrifuged for 5 min at 4,000 × *g* at 4°C. The supernatants were collected and protein concentration determined by a modified Bradford method (Bio-Rad Laboratories, Inc., Hercules, CA). Lysates were stored at −80°C.

### Tyrosine kinase receptor analysis

For analysis of tyrosine kinase receptor phosphorylation, a human PathScan® TKR signaling array was used according to the manufactures instructions (Cell Signaling, #7949, Danvers, MA). The TKR signaling array is a slide based antibody array that allows for the simultaneous detection of 28 tyrosine kinase receptor and 11 important signaling nodes when phosphorylated at tyrosine or other residues, 10 positive and two nonspecific IgG negative controls. Each TKR is spotted on the array in duplicates. A total of 150 μg of protein was used for each array pad. A biotinylated detection antibody cocktail followed by a streptavidin-conjugated DyLight 680® was used to visualize the bound detection antibody. Arrays were developed by fluorescent imaging and captured with a digital imaging system. Spot intensities were quantified using Typhoon™ 9410 and ImageQuant™ TL Array Analysis software version 7 (GE Healthcare, Piscataway, NJ). Mean fluorescence intensities for each TKR and signaling node were determined from the 12 cpAC and normal lung tissue lysates.

### Statistical analysis

Differences in the RT-PCR relative tumor and normal lung tissue cDNA densitometry values for the paired samples from three dogs and IHC pixel analyses values from all 12 dogs were assessed using a Wilcoxon matched-pairs signed- rank test for nonparametric analysis. Likewise, mean fluorescence intensities for each TKR and signaling node from the 12 dogs provided a small data set thus giving little power to detect non-Gaussian distributions and therefore were evaluated using a nonparametric analysis of Wilcoxon matched-pairs signed- rank test which assumes that the differences in groups are distributed symmetrically around their median. The confidence level was set at 95%. Significance was set at *P* < 0.05. Tests were executed with Prism® v.6.02 for Windows (GraphPad Prism®, La Jolla, CA).

## Abbreviations

ALK: Anaplastic lymphoma receptor tyrosine kinase; TIE-2: Angiopoietin-2; cpAC: Canine pulmonary adenocarcinoma; CD117/c-kit: Canine stem cell factor receptor; EML4: Echinoderm microtubule-associated protein-like 4; EGFR: Epidermal growth factor receptor; EphB1: EPH receptor B1; EphB3: EPH receptor B3; FGFR: Fibroblast growth factor receptor; hpAC: Human pulmonary adenocarcinomas; hNSCLC: Human non-small cell lung cancer; IHC: Immunohistochemistry; MAPK: Mitogen-activated protein kinase; MET: Met proto-oncogene; PFS: Progression-free survival; RT-PCR: Reverse-transcriptase polymerase chain reaction; ROS1: Tyrosine kinase domain: c-ros oncogene 1; RET: Receptor tyrosine kinase: ret proto-oncogene; TKIs: Tyrosine kinase inhibitors; phosphoTKR: Phosphorylated tyrosine kinase receptors; PDGFRα: Platelet-derived growth factor receptor alpha; PDGFRβ: Platelet-derived growth factor receptor beta; VEGFR2: Vascular endothelial growth factor receptor two; VEGF: Vascular endothelial growth factor; HER2: v-erb-b2 erythroblastic leukemia viral oncogene homolog 2; AKT: v-akt murine thymoma viral oncogene homolog.

## Competing interests

The authors declare no financial or non-financial competing interests.

## Authors’ contributions

GL conceived the study, designed the experiments, interpreted the data and wrote the manuscript. EM performed the experiments and data analysis and interpretation of the results as part of the NIH/Merial OSU veterinary summer scholar research program. CP performed the subjective evaluation of the histopathology. All authors read and approved the final manuscript.
